# Under Pressure: The Chronic Effects of Lower-Body Compression Garment Use during a 6-Week Military Training Course

**DOI:** 10.3390/ijerph19073912

**Published:** 2022-03-25

**Authors:** David T. Edgar, Christopher Martyn Beaven, Nicholas D. Gill, Matthew W. Driller

**Affiliations:** 1 Faculty of Health, Sport and Human Performance, University of Waikato, Hamilton 3240, New Zealand; goldmine@actrix.co.nz (D.T.E.); martyn.beaven@waikato.ac.nz (C.M.B.); nicholas.gill@nzrugby.co.nz (N.D.G.); 2 New Zealand Defence Force, Joint Support Group, Trentham Camp, Wellington 5019, New Zealand; 3 Sport and Exercise Science, School of Allied Health, Human Services and Sport, La Trobe University, Melbourne 3083, Australia

**Keywords:** recovery, physical training, performance, blood-flow, soreness, DOMS

## Abstract

Background: Previous studies have shown that compression garments may aid recovery in acute settings; however, less is known about the long-term use of compression garments (CG) for recovery. This study aimed to assess the influence of wearing CG on changes in physical performance, subjective soreness, and sleep quality over 6 weeks of military training. Methods: Fifty-five officer-trainees aged 24 ± 6 y from the New Zealand Defence Force participated in the current study. Twenty-seven participants wore CG every evening for 4–6 h, and twenty-eight wore standard military attire (CON) over a 6-week period. Subjective questionnaires (soreness and sleep quality) were completed weekly, and 2.4 km run time-trial, maximum press-ups, and curl-ups were tested before and after the 6 weeks of military training. Results: Repeated measures ANOVA indicated no significant group × time interactions for performance measures (*p* > 0.05). However, there were *small* effects in favour of CG over CON for improvements in 2.4 km run times (*d* = −0.24) and press-ups (*d* = 0.36), respectively. Subjective soreness also resulted in no significant group × time interaction but displayed *small* to *moderate* effects for reduced soreness in favour of CG. Conclusions: Though not statistically significant, CG provided *small* to *moderate* benefits to muscle-soreness and *small* benefits to aspects of physical-performance over a 6-week military training regime.

## 1. Introduction

Military training involves periods of high-intensity exercise interspersed with long periods of low-intensity on-feet activity with minimal recovery time [1,2,3]. Isometric and eccentric muscle contractions, essential in lifting and carrying during military training, frequently result in soreness and loss of function which can take several days to recover from [4,5], negatively affecting operational readiness in the military [6]. Military members are required to maintain a high level of operational performance throughout multiple daily and weekly training phases and while on deployments [2]. Those who recover faster from one day to the next may be able to tolerate higher loads and operate more effectively [7]. Thus, similar to sport and athletic settings, military environments represent an important working environment where recovery interventions and strategies could be implemented to assist day-to-day recovery.

Down-time and recovery periods may be maximized via the use of recovery interventions, such as compression garments (CG), which may support performance recovery following exercise [8,9], and perceptions of recovery [10,11]. Although reviews and meta-analyses on the use and benefits of CG have returned mixed findings [12,13,14], the majority of the literature supports the use of CG following exercise as an acute recovery strategy. A meta-analysis by Brown et al. [12] reported that CG may aid in the recovery following resistance training, eccentric loads, and aerobic activities. However, although the acute use of CG has been well documented, what is less known is the effect of chronic CG use in the exercise setting over a number of weeks. Of note, emerging research has suggested the potential for some recovery interventions (e.g., cold water immersion [CWI], non-steroidal anti-inflammatories [NSAIDs]) to have a blunting effect on the physiological adaptations and subsequent physical performance [15,16].

To our knowledge, only one study has evaluated the chronic use of CG in a long-term setting. A crossover study by Hu et al. [17] utilised CG for 4–5 h daily in male novice runners and concluded that CG may help to protect against overtraining based on heart rate variability data. However, the study did not investigate any performance-related outcomes to enable any conclusions relating to supporting or attenuating the adaptive process. Indeed, an emerging theory that warrants consideration when using recovery strategies that aim to mitigate inflammation, such as CG [15], is that over the long term, they may blunt molecular mechanisms associated with physiological adaptation [18]. Specifically, CWI protocols have been reported to suppress myofibrillar protein synthesis rates [19,20] and have been shown to attenuate the hypertrophy of type II muscle fibres [21]. Therefore, the notion that CG will support long-term adaptation may need to be reconsidered, given the possibility of a reduced physiological stimulus. Hydrostatic pressure from CWI and elastic pressure from CG may enhance blood flow and fluid movement required for nutrient and metabolite delivery to working muscles, while reducing inflammation [15,22,23]. It is therefore plausible that this process may blunt signalling pathways following exercise, potentially impacting on adaptive strength and power development [15]. Whether similar effects of chronic CWI on performance adaptations could translate to CG recovery is yet to be realised.

Despite the plethora of research on acute recovery strategies in athlete settings, the benefit of longer periods of CG wear to aid recovery and subsequent performance is relatively unknown. As has emerged in CWI research, there is a possibility that if CG reduce the underlying stimulus for adaptation, their long-term use could potentially blunt the physiological adaptations to training [15,18]. Therefore, the aim of the current study was to determine the effect of CG on physical performance and subjective muscle soreness when worn daily for 4–6 h in the evening, for the duration of a 6-week training course in the military setting.

## 2. Materials and Methods

### 2.1. Participants

A total of 55 healthy officer trainees (44 male/11 female) aged 24 ± 6 y, from the New Zealand Defence Force (NZDF) Joint Officer Induction Course (JOIC), participated in the current study. Participation in the study was voluntary with inclusion dependent on passing the pre-course medical examination. Trainees’ data were to be excluded if they withdrew voluntarily from the course or were medically removed. Ethical approval for the study was obtained from an institutional Human Research Ethics Committee (HREC) (Health) #2018-0. Sample-size calculations based on an a priori two-sided alpha of 0.05, 0.8 power, and informed by minimal detectable change and variability data from Edgar et al. (2021) regarding the response variables, indicated a sample size requirement of 24 (press-ups), to 52 (curl-ups), and 54 (2.4 km run).

### 2.2. Experimental Design

The experimental design included a randomised, parallel-group intervention study, whereby participants were split into two groups: compression garments (CG, *n* = 27) and control (no compression [CON] *n* = 28). All participants were tested on their physical performance before and after 6weeks of officer training. Physical performance data were collected in Weeks One and Six during two 90-min sessions, with subjective wellbeing monitoring (muscle soreness and sleep quality) completed weekly at the conclusion of each week’s training.

### 2.3. Compression Garments 

Sports compression tights with stirrups (2XU, Melbourne, Australia) were used in the current study. The 2XU MCS (muscle containment stamping) compression tights were made from PWX 105D INVISTA LYCRA ^®^FIBER fabric, and consisted of 65% nylon (140 denier) and 35% elastane (360 denier). The garments were graded with a level II compression rating from the manufacturer, defined by a pressure range of 23–32 mmHg [24]. All CG were individually sized following the manufacturer guidelines, based on stature and body mass, and ran from the medial malleolus of the ankle (incorporating the midfoot in a stirrup) to superior to the iliac crest. The CG were required to be worn for a period of 4–6 h every evening, but not for longer than 6 h, and were required to be removed before going to bed. For the full duration of the 6-week course, CG were worn every evening apart from nights spent on field exercise (one night in Week 3, and three nights in Week 5). All participants complied and wore CG for all nights required. Therefore, the CG group wore the compression for a total of 32 nights throughout the study. Two sets of CG were issued to each trainee to allow for daily rotation of washing and for hygiene purposes.

### 2.4. Compression Garment Pressure Monitoring

The applied pressure of the CG was tested using a Kikuhime pressure monitoring device (MediGroup, Melbourne, Australia) at the medial malleolus of the ankle, and at the mid-point and maximal circumference of the calf and thigh. These landmarks are commonly used when measuring the pressure of full-length compression garments [10,25]. Garment pressure measurements were taken in the first week of the course on Day 3, and in the last week of the course (Week 6, Day 5). The Kikuhime pressure monitor has been shown to be a valid (ICC = 0.99, CV = 1.1%) and reliable (CV = 4.9%) tool for compression measurement in athlete settings [26].

### 2.5. Subjective Monitoring

During the study period, trainees completed a psychological questionnaire at the completion of each week on a Monday morning in a class prior to the next week commencing, as used previously [27]. The questionnaire was individually completed via a paper-based form, and assessed each trainee’s general muscle soreness and sleep quality on a five-point scale of 1 to 5 with 0.5 point increments (5 = very good, 4 = good, 3 = normal, 2 = poor, 1 = very poor). Muscle soreness and sleep quality were included in the current study, as previous research has shown that less soreness (via the use of recovery interventions) may lead to improved sleep [28].

### 2.6. Physical Training Program

Physical training (PT) comprised a controlled two-week introduction phase of body weight exercises and aerobic conditioning. In Weeks 3 and 4, the intensity of PT increased to challenge individuals. Week 5 and Week 6 then focused on functional fitness and conditioning. This progressive training included increased load carriage with a combination of field packs, day packs, webbing, and weapons. A total of 18 × 90-min periods were allocated to physical training over the 6-week period and included a combination of aerobic interval running, strength training, circuits, swimming, and bike–boxing–rowing intervals as outlined in Table 1.

### 2.7. Fitness Testing

The standard NZDF JOIC fitness evaluation was conducted by NZDF Physical Training Instructors (PTIs) before and after the course. This evaluation consisted of three key components: (1) 2.4 km time-trial road run, (2) maximum curl-ups (also known as sit-ups), and (3) maximum press-ups conducted on a wooden gym floor. These tests have previously been used in the military population to provide an evaluation of fitness [1]. Fitness testing was conducted at 9:00 a.m. with identical morning routines prior to each testing session. Run times were measured via stopwatch to the nearest second by a designated PTI. Press-ups and curl-ups repetitions were counted by a PTI every time the full range of motion was completed, maintaining a consistent tempo, until failure. For both the press-ups and curl-ups, one warning was given for an incomplete repetition, prior to fatigue or participants being stopped by the PTI [1].

### 2.8. Statistical Analysis

Descriptive statistics are shown as mean ± SD values unless stated otherwise. All statistical analyses were performed using the Statistical Package for Social Science (V. 22.0, SPSS Inc., Chicago, IL, USA), with statistical significance set at *p* ≤ 0.05. To examine whether there were any performance differences between groups, a 2 × 2, Group (CG and CON) × Time (pre and post) repeated measures ANOVA was conducted, and interactions were assessed. For soreness and sleep, a repeated measures ANOVA 2 × 6 Group (CG and CON) × Time (Week 1, Week 2, Week 3, Week 4, Week 5, and Week 6) interaction was performed on weekly scores between groups. A Bonferroni adjustment was applied if significant main effects were detected. Analysis of the distribution of residuals was verified visually with histograms and also using the Shapiro–Wilk test of normality. Magnitudes of the standardized effects between pre and post scores between groups were calculated using Cohen’s *d* and interpreted using thresholds of 0.2, 0.5, and 0.8 for *small*, *moderate*, and *large*, respectively [29]. Effects were deemed unclear if the 90% confidence intervals overlapped the thresholds for the smallest worthwhile change (*d* > 0.2).

## 3. Results

From trainees who began the study (*n* = 56), one from the CG did not complete the study due to withdrawing from military training, with final group allocation: CG (*n* = 27) and CON (*n* = 28). The applied pressure of the CG at commencement was 25.4 mmHg at the ankle, 21.6 mmHg at the calf, and 15.0 mmHg at the thigh, indicating the average CG profile was graduated (Table 2). There was a significant decrease in CG pressure from pre to post training course (Table 2).

**Figure 1:**
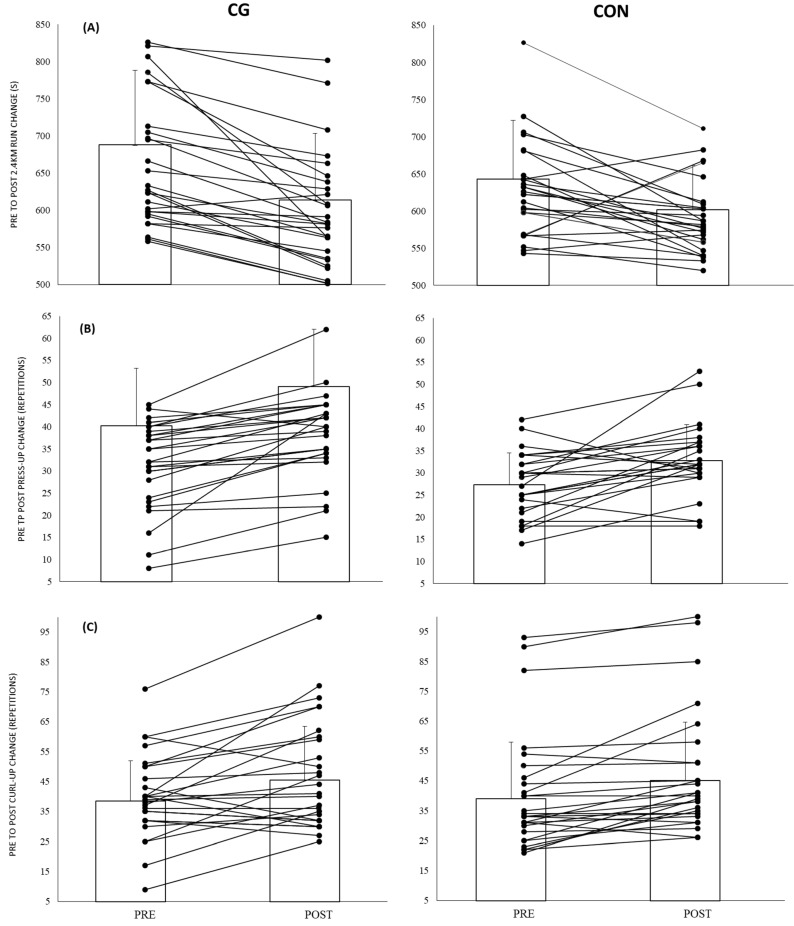
Pre to post performance change for compression garments (CG) and control (CON) groups over the 6 weeks of military training. Boxed columns indicate mean and SD, and lines indicate individual participant results. (**A**) 2.4 km run (s), (**B**) press-ups (repetitions), (**C**) curl-ups (repetitions).

The ANOVA detected no significant Group × Time interaction for performance measures; however, *small* effects were observed in favour of CG for run and press-up performance (Table 3). Specifically, the CG group improved 18 s more than the CON group (46.8 vs. 28.9 s, *d* = −0.24, *small*) in the 2.4 km run, and by 3.7 more repetitions (5.2 vs. 1.5 repetitions; *d* = 0.36, *small*) in the press-ups (Figure 1).

**Figure 2:**
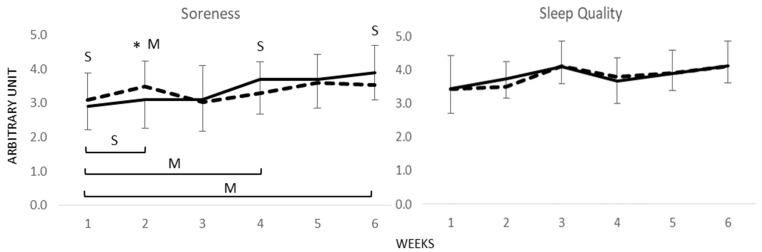
Mean ± SD comparison for weekly subjective muscle soreness and sleep quality for compression garments (GC) (bold line) and CON (dashed line), for the duration of 6 weeks of military training. * Significant difference between group values (*p* < 0.05), M: *moderate* Cohen’s *d* effect (*d* > 0.5), S: *small* Cohen’s *d* effect (*d* > 0.2).

When comparing subjective soreness data for the 6-week training period, a significant and *moderate* effect (*p* < 0.01, *d* = 0.77) was observed in favour of CG in Week 2. Non-significant but *moderate* effects were observed for CG for change over time from Week One to Week Four (*p* = 0.08, *d* = −0.79), and Week One to Week Six (*p* = 0.18, *d* = −0.67); however, only *small* differences were observed in Weeks One, Four, and Six (Figure 2). Subjective sleep quality displayed no significant Group × Time interaction (*p* = 0.59), with only *trivial* effects between groups (*d* = −0.14, Figure 2). No significant or meaningful differences in sleep quality were observed at any time point (*p* > 0.05; *d =* 0.01 to −0.29).

## 4. Discussion

The aim of this study was to determine the effect of CG use on physical performance, subjective muscle soreness, and sleep quality over 6 weeks of military training. Findings suggest that, although not statistically significant, wearing lower body CG for 4–6 h each evening elicited *small* improvements in 2.4 km run time and press-up performance. Furthermore, there were significant perceptual benefits, with *moderate* reductions in muscle soreness in the CG group. No differences in subjective sleep quality were observed at any time during the 6-week intervention period. We can conclude from these results, that CG were not detrimental to physical performance when used in the chronic setting, and therefore, it is unlikely they had a negative impact on the adaptive response to training, as has been seen in CWI studies [15,30,31].

To our knowledge, no research to date has evaluated the daily use of CG over an extended time frame (e.g., more than 2 weeks), with most studies investigating the acute application of CG immediately post-exercise. Hu et al. [17] found that application of CG over a 2-week period contributed to improved HRV markers of recovery in amateur runners, with the authors concluding that this may have been due to increased venous return and decreased venous pooling; reducing swelling and muscle soreness. In the current study, although not significant, changes observed provided *small* effects in favour of CG for run and press-up performance. Significant and *moderate* positive effects were observed for subjective muscle soreness at various time points. Our findings suggest that the CG group may have perceived that they recovered better and, potentially, were able to manage and maintain a greater training load than the CON group throughout the course.

The *small* effect sizes for aerobic fitness and upper body muscular endurance in favour of CG are not congruent with recent literature that would suggest the chronic use of recovery interventions may in fact blunt adaptation. With specific reference to CWI, either negligible or negative effects on performance have been reported [30,31]. However, in an elite athlete setting, Halson et al. [32] reported that chronic CWI use in endurance trained cyclists, similar to the current study, allowed performance and perceptual recovery to be better maintained in the CWI group when compared with CON. Similarly, Tavares et al. [33] investigated the effects of daily chronic CWI during a 3-week pre-season period (12 days in total) on elite rugby players, and found that chronic use of CWI supported a *moderate* beneficial effect on muscle soreness when compared with a control group. Both Halson et al. [32] and Tavares et al. [33] did not report any detrimental effects to performance, but suggested long term benefits to adaptation and reducing fatigue and soreness from chronic application of CWI in the specific context of athletes with high training volumes and density. Thus, the use of recovery intervention is especially important given that the early recovery phase involves the overlapping process of inflammation and the occurrence of secondary muscle damage [22] that will negatively impact on subsequent training and adaptive stimulus.

The current study displayed significant *moderate* effects for subjective perceptions of muscle soreness in favour of CG. These findings draw parallels with the Roberts et al. [19] investigation on CWI, where it was outlined that central perceptions of better recovery may play a more dominant role than peripheral physiological factors in the capacity for athletes to recover from exercise. Our findings also align closely with the meta-analyses of Born et al. [34], which generally showed improved perception of muscle soreness with the use of CG in the acute setting. A potential link associated with reduced muscle soreness is sleep quality, and although it has been hypothesized that sleep may be impaired with high levels of soreness [35], the current study displayed no difference between CG and CON groups for sleep quality over the 6-week course.

Mechanisms associated with improved recovery from physical exercise when wearing CG are not fully understood and warrant further investigation [15,36]. Limitations from the current study included only monitoring weekly soreness, and the number of performance measures assessed. Daily soreness measures may have provided a clearer indication of muscle soreness fluctuations around specific training days and activities. Ideally, future work would assess mechanistic measures to investigate mitochondrial biogenesis adaptations (e.g., PGC-1α mRNA and p-AMPK) [15], to provide a physiological underpinning explaining the beneficial effects of the chronic use of CG. Consideration should also be given to the finding that CG fabrics lose integrity/pressure over time with regular wear, which may reduce their efficacy—as noted in the current study, with the applied pressure decreasing over the course of the study. New garments provided halfway through the course may have prevented the observed reduction in pressure profiles and may have impacted on the study findings. However, we feel the current study is more likely to replicate what happens in the real-world environment where many consumers only own a single pair of CG, therefore increasing the ecological validity of the study.

## 5. Conclusions

The chronic wearing of CG might provide a viable recovery strategy in the situations where high volumes of strength and endurance training are performed with limited recovery time. In our applied military context, the chronic use of CG allowed military personnel to cope with high training loads and mitigate muscle soreness more effectively during intense training courses. There was no evidence to suggest a detrimental effect on overall adaptation, with small but meaningful improvements in physical capacity associated with wearing CG.

## Figures and Tables

**Table 1:** Joint officer induction course physical and military training program outline.

Variation	Activity	Duration	Number of Sessions Per Week						
	Physical Training		Wk 1	Wk 2	Wk 3	Wk 4	Wk 5	Wk 6	Total
**1**	Aerobic Interval Running	90 min	1	1	1	1	1		**5**
**2**	Circuit Training (Strength Endurance)	90 min	1	1	1	1	1		**5**
**3**	Swimming/Pool Circuit	90 min	1	1	1	1		1	**5**
**4**	Stretch, Mobility and Recovery Flush	90 min	1	1		1		1	**4**
	Military Training								
**1**	Drill (Parade Ground)	30–60 min	3	2	3	2	2	3	**15**
**2**	Weapons Training	4 h+	1	4	3	2	2	3	**15**
**3**	Land Navigation	3–6 h		1	2	1			**4**
**4**	Sea Survival	24 h					1		**1**
**5**	Bush Craft	6 h		1	1	1			**3**
**6**	Tactical Field Exercise	5 days						1	**1**
**Weekly Total**			**8**	**12**	**12**	**10**	**7**	**9**	

Note: A ten minute 6:00 a.m. early morning activity (EMA) was also conducted daily including stretching, mobility, and cognitive reaction games.

**Table 2:** Applied pressure measurements (mean ± SD [mmHg]) for full length lower limb compression garment at the ankle, calf, and thigh. Pre to post 6 weeks of military training.

	Pre (mmHg)	Post (mmHg)	Change (mmHg)	*p*-Value
Ankle	25.4 ± 2.1	19.7 ± 1.8	−5.7 ± 1.9	<0.01
Calf	21.6 ± 2.8	18.3 ± 1.9	−3.3 ± 1.9	=0.01
Thigh	15.0 ± 4.3	11.8 ± 3.1	−3.2 ± 2.0	<0.01

**Table 3:** Mean ± SD comparison for pre to post 2.4 km run, press-ups, and curl-ups, for the compression garment (CG, *n* =27) and control (CON, *n* = 28) groups, including group interaction *p*-value, *d*-value, and effect size descriptor (in italics) for the duration of 6 weeks of military training.

					Group × Time Interaction	
		Pre	Post	Change	*p*-Value	ES (*d*) ± 90% CI
2.4 km Run (s)						
	CG	650.5 ± 88.7	603.7 ± 79.5	−46.8 ± 66.1	0.284	0.24 ± 0.36, *Small*
	CON	620.7 ± 70.1	591.8 ± 55.2	−28.9 ± 53.3		
Press-Ups (repetitions)						
	CG	31 ± 10	36 ± 10	5 ± 8	0.171	0.36 ± 0.43, *Small*
	CON	28 ± 7	29 ± 10	1 ± 10		
Curl-Ups (repetitions)						
	CG	37 ± 13	41 ± 18	4 ± 18	0.521	0.19 ± 0.50, *Trivial*
	CON	40 ± 20	41 ± 17	1 ± 20		

## Data Availability

Data supporting reported results is available from David Edgar; David.Edgar@nzdf.mil.nz.

## References

[1] Edgar D., Gill N., Driller M. (2020). Physical characteristics of New Zealand Army, Navy and Airforce officer trainees’over a 6-week joint officer induction course. J. Sport Exerc. Sci..

[2] Knapik J., Redmond J., Grier T., Sharp M. (2018). Secular trends in the Physical Fitness of United States Army Infantry Units and Infantry Soldiers, 1976–2015. Mil. Med..

[3] Orr R., Pope R. (2015). Optimizing the physical training of military trainees. Strength Cond. J..

[4] Harty P., Cottet M., Malloy J., Kerksick C. (2019). Nutritional and supplementation strategies to prevent and attenuate exercise-induced muscle damage: A brief review. Sports Med.-Open.

[5] Howatson G., Van Someren K., Hortobagyi T. (2007). Repeated bout effect after maximal eccentric exercise. Int. J. Sports Med..

[6] Orr R., Pope R., Johnston V., Coyle J. (2014). Soldier occupational load carriage: A narrative review of associated injuries. Int. J. Inj. Control. Saf. Promot..

[7] Vartanian O., Fraser B., Saunders D., Ralph C., Lieberman H., Morgan C., Cheung B. (2018). Changes in mood, fatigue, sleep, cognitive performance and stress hormones among instructors conducting stressful military captivity survival training. Physiol. Behav..

[8] Argus C., Driller M., Ebert T., Martin D., Halson S. (2013). The effects of 4 different recovery strategies on repeat sprint-cycling performance. Int. J. Sports Physiol..

[9] Marqués-Jiménez D., Calleja-González J., Arratibel I., Delextrat A., Terrados N. (2016). Are compression garments effective for the recovery of exercise-induced muscle damage? A systematic review with meta-analysis. Physiol. Behav..

[10] Atkins R., Lam W., Scanlan A., Beaven M., Driller M. (2020). Lower-body compression garments worn following exercise improves perceived recovery but not subsequent performance in basketball athletes. J. Sports Sci..

[11] Driller M., Halson S. (2013). The effects of lower-body compression garments on recovery between exercise bouts in highly-trained cyclists. J. Sci. Cycl..

[12] Brown F., Gissane C., Howatson G., Van Someren K., Pedlar C., Hill J. (2017). Compression garments and recovery from exercise: A meta-analysis. Sports Med..

[13] da Silva C., Helal L., da Silva R., Belli K., Umpierre D., Stein R. (2018). Association of lower limb compression garments during high-intensity exercise with performance and physiological responses: A systematic review and meta-analysis. Sports Med..

[14] Lee D., Ali A., Sheridan S., Chan D., Wong S. (2020). Wearing compression garment enhances central hemodynamics? A systematic review and meta-analysis. J. Strength Cond. Res..

[15] Broatch J., Petersen A., Bishop D. (2018). The influence of post-exercise cold-water immersion on adaptive responses to exercise: A review of the literature. Sports Med..

[16] Lundberg T., Howatson G. (2018). Analgesic and anti-inflammatory drugs in sports: Implications for exercise performance and training adaptations. Scand. J. Med. Sci. Sports.

[17] Hu J., Browne J., Baum J., Robinson A., Arnold M., Reid S., Neufeld E., Dolezal B. (2020). Lower limb graduated compression garments modulate autonomic nervous system and improve post-training recovery measured via heart Rate variability. Int. J. Exerc. Sci..

[18] Earp J., Hatfield D., Sherman A., Lee E., Kraemer W. (2019). Cold-water immersion blunts and delays increases in circulating testosterone and cytokines post-resistance exercise. Eur. J. Appl. Physiol..

[19] Roberts L., Nosaka K., Coombes J., Peake J., Peake J. (2014). Cold water immersion enhances recovery of submaximal muscle function following resistance exercise. Am. J. Physiology. Regul. Integr. Comp. Physiol..

[20] Fuchs C., Kouw I., Churchward-Venne T., Smeets J., Senden J., Lichtenbelt W., Verdijk L., van Loon L. (2020). Postexercise cooling impairs muscle protein synthesis rates in recreational athletes. J. Physiol..

[21] Fyfe J., Broatch J., Trewin A., Hanson E., Argus C., Garnham A., Halson S., Polman R., Bishop D., Petersen A. (2019). Cold water immersion attenuates anabolic signaling and skeletal muscle fiber hypertrophy, but not strength gain, following whole-body resistance training. J. Appl. Physiol..

[22] Chazaud B. (2020). Inflammation and skeletal muscle regeneration: Leave it to the macrophages!. Trends Immunol..

[23] Lee D., Law H., Ali A., Sheridan S., Wong S., Lee S. (2020). Compression garment-induced leg changes increase hemodynamic responses in healthy individuals. Int. J. Sports Med..

[24] Kennzeichnung D. (2000). Medizinische Kompressionsstrumpfe Deutsches Institut Fur Gutesicherung UND Kennzeichnung.

[25] Dascombe B., Hoare T., Sear J., Reaburn P., Scanlan A. (2011). The effects of wearing undersized lower-body compression garments on endurance running performance. Int. J. Sports Physiol. Perform..

[26] Brophy-Williams N., Driller M., Halson S., Fell J., Shing C. (2014). Evaluating the Kikuhime pressure monitor for use with sports compression clothing. Sports Eng..

[27] Edgar D., Gill N., Beaven C., Zaslona J., Driller M. (2021). Sleep duration and physical performance during a 6-week military training course. J. Sleep Res..

[28] Halson S. (2013). Sleep and the elite athlete. Sports Sci. Exch..

[29] Cohen J. (1988). Statistical Power Analysis for the Behavioral Sciences.

[30] Roberts L., Raastad T., Markworth J., Figueiredo V., Egner I., Shield A., Cameron-Smith D., Coombes J., Peake J. (2015). Post-exercise cold water immersion attenuates acute anabolic signalling and long-term adaptations in muscle to strength training. J. Physiol..

[31] Yamane M., Ohnishi N., Matsumoto T. (2015). Does regular post-exercise cold application attenuate trained muscle adaptation. Int. J. Sports Med..

[32] Halson S., Bartram J., West N., Stephens J., Argus C., Driller M., Sargent C., Lastella M., Hopkins W., Martin D. (2014). Does hydrotherapy help or hinder adaptation to training in competitive cyclists. Med. Sci. Sport Exerc..

[33] Tavares F., Beaven M., Teles J., Baker D., Healey P., Smith T., Driller M. (2019). Effects of chronic cold-water immersion in elite rugby players. Int. J. Sports Physiol. Perform..

[34] Born D., Sperlich B., Holmberg H. (2013). Bringing light into the dark: Effects of compression clothing on performance and recovery. Int. J. Sports Physiol. Perform..

[35] Leeder J., Gissane C., van Someren K., Gregson W., Howatson G. (2012). Cold water immersion and recovery from strenuous exercise: A meta-analysis. Br. J. Sports Med..

[36] Hettchen M., Glöckler K., von Stengel S., Piechele A., Lötzerich H., Kohl M., Kemmler W. (2019). Effects of compression tights on recovery parameters after exercise induced muscle bamage: A randomized controlled crossover study. Evid. Based Complementary Altern. Med..

